# A circuit view of deep brain stimulation in Alzheimer’s disease and the possible mechanisms

**DOI:** 10.1186/s13024-019-0334-4

**Published:** 2019-08-08

**Authors:** Danfang Yu, Huanhuan Yan, Jun Zhou, Xiaodan Yang, Youming Lu, Yunyun Han

**Affiliations:** 1 Department of Neurobiology, School of Basic Medicine and Tongji Medical College, Huazhong University of Science and Technology, Wuhan, China; 2 Department of Neurology, Provincial Hospital of Integrated Chinese & Western Medicine, Wuhan, China; 3 Department of Physiology, School of Basic Medicine and Tongji Medical College, Huazhong University of Science and Technology, Wuhan, China; 4 Institute for Brain Research, Collaborative Innovation Center for Brain Science, Huazhong University of Science and Technology, Wuhan, China; 5 Biomedical Engineering Department, Huazhong University of Science and Technology, Wuhan, China

**Keywords:** Alzheimer’s disease, Deep brain stimulation, Fornix, Entorhinal cortex, Nucleus basalis of Meynert, Vertical limb of diagonal band

## Abstract

Alzheimer’s disease (AD) is characterized by chronic progressive cognitive deterioration frequently accompanied by psychopathological symptoms, including changes in personality and social isolation, which severely reduce quality of life. Currently, no viable therapies or present-day drugs developed for the treatment of AD symptoms are able to slow or reverse AD progression or prevent the advance of neurodegeneration. As such, non-drug alternatives are currently being tested, including deep brain stimulation (DBS). DBS is an established therapy for several neurological and psychiatric indications, such as movement disorders. Studies assessing DBS for other disorders have also found improvements in cognitive function, providing the impetus for clinical trials on DBS for AD. Targets of DBS in AD clinical trials and animal model studies include the fornix, entorhinal cortex (EC), nucleus basalis of Meynert (NBM), and vertical limb of diagonal band (VDB). However, there is still no comprehensive theory explaining the effects of DBS on AD symptoms or a consensus on which targets provide optimal benefits. This article reviews the anatomy of memory circuits related to AD, as well as studies on DBS rescue of AD in these circuits and the possible therapeutic mechanisms.

## Background

Alzheimer’s disease (AD) represents a considerable health threat as increases in life expectancy elevate the incidence of AD worldwide [1]. It is characterized by cognitive decline, alterations in synaptic transmission, and neuronal death brought on by the accumulation of neurofibrillary tangles in neurons and neuritic amyloid beta (Aβ) plaque in the brain parenchyma [2]. Currently, there is no viable therapy to slow or reverse AD progression. Furthermore, no present-day drugs developed for the treatment of AD symptoms can prevent the underlying progression of neurodegeneration [3]. Therefore, several non-pharmaceutical approaches are currently being tested [4], including deep brain stimulation (DBS) [5].

As an established therapy for the treatment of medically refractory movement disorders, such as Parkinson’s disease [6], essential tremor [7], and dystonia [8], the benefits of DBS are well documented. The minimal invasiveness and relatively low levels of serious complications have also led to its application in other neurological and psychiatric disorders, such as depression [9], obsessive-compulsive disorder [10], Tourette syndrome [11], obesity [12], and chronic pain [13].

The initiation of clinical DBS trials in AD came from the fortuitous discovery of cognitive improvements in patients undergoing DBS for other diseases, including obesity [14] and Parkinson’s disease [15]. Such studies also highlighted potential experimental target areas, with common DBS targets for AD treatment found to include the fornix [5], entorhinal cortex (EC) [16], nucleus basalis of Meynert (NBM) [17], vertical limb of diagonal band (VDB) [18], and ventral capsule/ventral striatum [19]. However, it remains uncertain which of these targets is most effective or how chronic stimulation impacts neurocellular and circuit function. Given the multiplicity of effective stimulation targets, the question naturally arises as to whether their therapeutic mechanisms are similar. In other words, it is unclear if the compensatory network processes enabled by DBS are similar or unique for each target. This issue is critical for effectively targeting different AD symptoms and pathogenic mechanisms. This article anatomy of memory circuits related to AD, as well as studies on DBS rescue of AD in these circuits and the possible therapeutic mechanisms, with particular emphasis on the medial temporal lobe (fornix and EC) and basal forebrain (BF) cholinergic system (NBM and VDB).

## Main text

### Relationship between memory circuitry and AD

#### Medial temporal lobe

The first reported symptoms of AD are difficulty in remembering new information and episodic memory loss [20], which are hippocampus dependent [21]. Hippocampal atrophy is widely observed in the majority of AD patients and is considered a characteristic physiological feature associated with cognitive deficits. Indeed, hippocampal atrophy imaging is a valuable tool for assessing disease stage and has provided multiple clues to the mechanisms underlying the behavioral manifestations of AD [22]. The first comprehensive hierarchical staging of AD by neuropathological examination revealed that the accumulation of neurofibrillary tangles occurs initially in the EC and then spreads to all isocortical association areas, including the hippocampal formation [23]. Consistent with the neuropathological literature, the presence of elevated neocortical (18) F-T807 (a Tau protein binding molecule that can be detected by positron emission tomography (PET)), particularly in the inferior temporal gyrus, is associated with clinical impairment [24].

As part of the Papez circuit, the fornices are thought to contribute to the efficient encoding and normal recall of new episodic information [25]. Indeed, forniceal lesions produce severe memory impairments [26]. Quantitative fiber tracking has demonstrated an age-dependent reduction in forniceal integrity during healthy aging [27] and atrophy of the fornix and mammillary body (MB) can accompany the change from mild cognitive impairment to clinical AD [28]. A significant reduction in fractional anisotropy, which is indicative of reduced white matter tract integrity, is also found to precede forniceal volume loss in patients with genetically inherited dementia, as measured by diffusion tensor imaging [29].

#### Basal forebrain cholinergic system

Since 1906, when Alois Alzheimer first delineated the symptomatology of the disease that bears his name, many have tested the hypothesis that degeneration or dysfunction of BF cholinergic circuitry is responsible for the cognitive impairments associated with AD [30]. The first evidence for network dysfunction in AD was the NBM cholinergic neuronal loss found in postmortem brains [31]. These findings provided the foundation of the cholinergic hypothesis, which states that the loss of cholinergic neurons is a seminal event leading to AD [30]. Identification of neurofibrillary tangle formation and degeneration of NBM cholinergic neurons supports the cholinergic hypothesis [32]. Indeed, there is a strong correlation between BF cholinergic neuron survival and AD onset and severity [18, 33, 34]. BF neurons, specifically those in the NBM, selectively degenerate in AD [35]. Severe deficits in the cholinergic VDB-hippocampal projection system are also found in AD [36], in agreement with our finding that cholinergic fiber lesions appear earlier than cholinergic neuronal loss in AD model mice [34]. In AD mice, direct cholinergic synaptic transmission from the VDB to hippocampus is impaired, which contributes to the loss of pattern separation-dependent spatial memory [18].

### Anatomy of memory circuitry

#### Medial temporal lobe

**Fig. 1:**
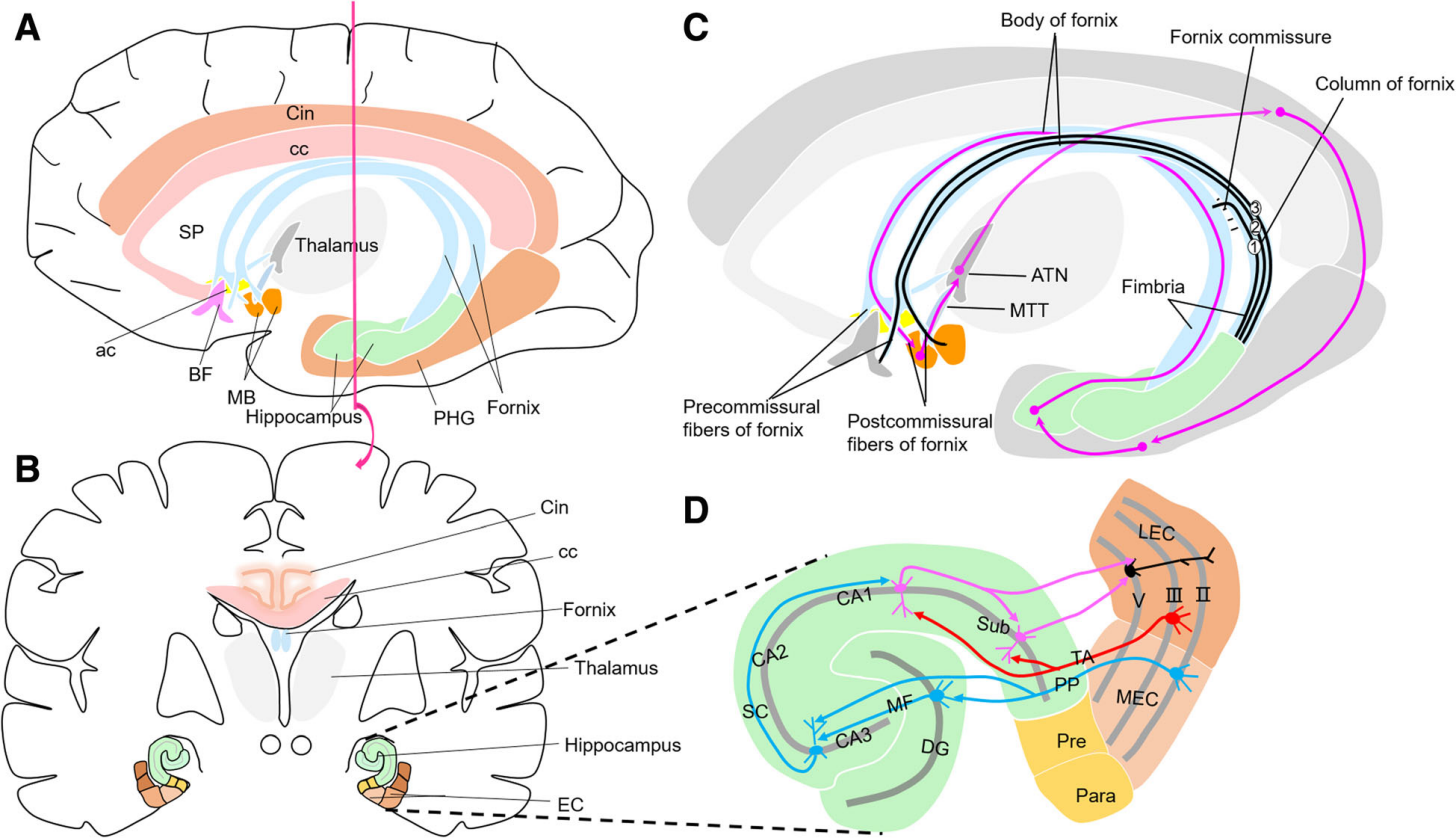
**a** Lateral-medial view showing location of the hippocampus and parahippocampus gyrus (PHG) in the temporal lobe. **b** Brain is sectioned coronally to show the hippocampus and entorhinal cortex (EC) of the medial temporal lobe. **c** Anatomy of the fornix and Papez circuit. Black lines show columns of the fornix. Column 1 = fibers crossing the hippocampal commissure to join the contralateral forniceal body. Column 2 = postcommissural fibers of the fornix that arise primarily from the subiculum and project to the mammillary body. Column 3 = precommissural fibers of the fornix that arise from the pyramidal cell layer of the hippocampus and project to the BF. Magenta lines indicate path of Papez circuit (hippocampus-fornix-MB-MTT-ATN-Cin-EC-hippocampus) and dots indicate integration sites. **d** Anatomy of EC-hippocampus network. Partial magnification of Fig. 1b with a 90° counterclockwise rotation. EC and hippocampus form a principally unidirectional network, with input from the EC. PP consists of axons from EC layer II stellate cells, projecting indirectly to the hippocampal CA1 area via Mossy fibers from the DG and Schaffer collaterals from the CA3 (red lines). TA consists of axons from EC layer III pyramidal cells, projecting directly to hippocampal CA1 (blue lines). Neurons from CA1 and subiculum (Sub), in turn, send the main hippocampal output back to the EC layer V (magenta lines), forming a loop. Abbreviations: ac, anterior commissure; ATN, anterior thalamic nuclei; BF, basal forebrain; cc, corpus callosum; Cin, cingulate gyrus; DG, dentate gyrus; EC, entorhinal cortex; LEC, lateral entorhinal cortex; MB, mammillary body; MEC, medial entorhinal cortex; MF, Mossy fibers; MTT, mammillothalamic tract; Para, parasubiculum; PHG, parahippocampal gyrus; PP, perforant path; Pre, presubiculum; Sub, subiculum; SC, Schaffer collateral; SP, septa pellucidum; TA, temporoammonic path

Major subdivisions of the medial temporal lobe cortex include the hippocampal formation and parahippocampal gyrus (Fig. 1) [37, 38]. The hippocampus can be described more holistically as a curved and recurved sheet of cortex that is folded onto the medial surface of the temporal lobe. The EC, located anteriorly in the parahippocampal gyrus, is the major input structure to the hippocampus, whereas the fornix is the major output structure.

The EC is divided into the medial EC (MEC) and lateral EC (LEC) based on distinctive cytoarchitecture and connectivity patterns [38]. The MEC contains strongly position-related (spatial) neurons [38–40], whereas the LEC contains neurons encoding object information, attention, and motivation [38, 40, 41]. The afferents from the EC to hippocampus are divided into the perforant and temporoammonic paths, also known as the indirect and direct paths, respectively, referring to their relay connections to the CA1 [21, 42] (see Fig. 1d). Axons from the LEC form the lateral perforant path, whereas those from the MEC form the medial perforant path to the hippocampus [43]. LEC projection neurons selectively form direct excitatory synapses onto a subpopulation of calbindin-expressing pyramidal neurons in the CA1, whereas MEC neurons uniformly innervate all CA1 pyramidal neurons [44].

The fornix (Fig. 1c) is a white matter tract running through the medial aspect of the cerebral hemispheres [45, 46]. The fornix is a bilateral structure with rich connections between both hemispheres. At the level of the foramen of Monro and anterior commissure, the body of the fornix splits in a way that reflects the areas of origin in the hippocampus. The fibers passing in front of the anterior commissure (precommissural fibers) are those originating from the pyramidal cell layer of the hippocampus (along with fibers from the EC and subiculum). The fibers passing behind the commissure (postcommissural fibers) arise from the subiculum of the hippocampus.

The precommissural fibers extend down the columns of the fornix to terminate in the BF (including the septum), ventral striatum, and prefrontal cortex. They also contain substantial projections from the septum and VDB to the hippocampus. The postcommissural fibers extend down the columns to the thalamus and hypothalamus. About one half to two thirds of these fibers directly innervate the anterior thalamic nucleus of the thalamus, with the remaining fibers descending to innervate the MB of the hypothalamus, among other regions.

First described by James Papez [47], the classic Papez circuit contains most of the medial temporal lobe, including the hippocampus, EC, and fornix (Fig. 1c). Some fibers of the fornix originate from the hippocampus and terminate in the MB. Fibers from the MB pass backward into the anterior thalamic nucleus via the mammillothalamic tract (MTT) and then onto the cingulum. The cingulum is connected to association cortices and the hippocampus, thus completing the Papez loop.

#### Basal forebrain cholinergic system

**Fig. 2:**
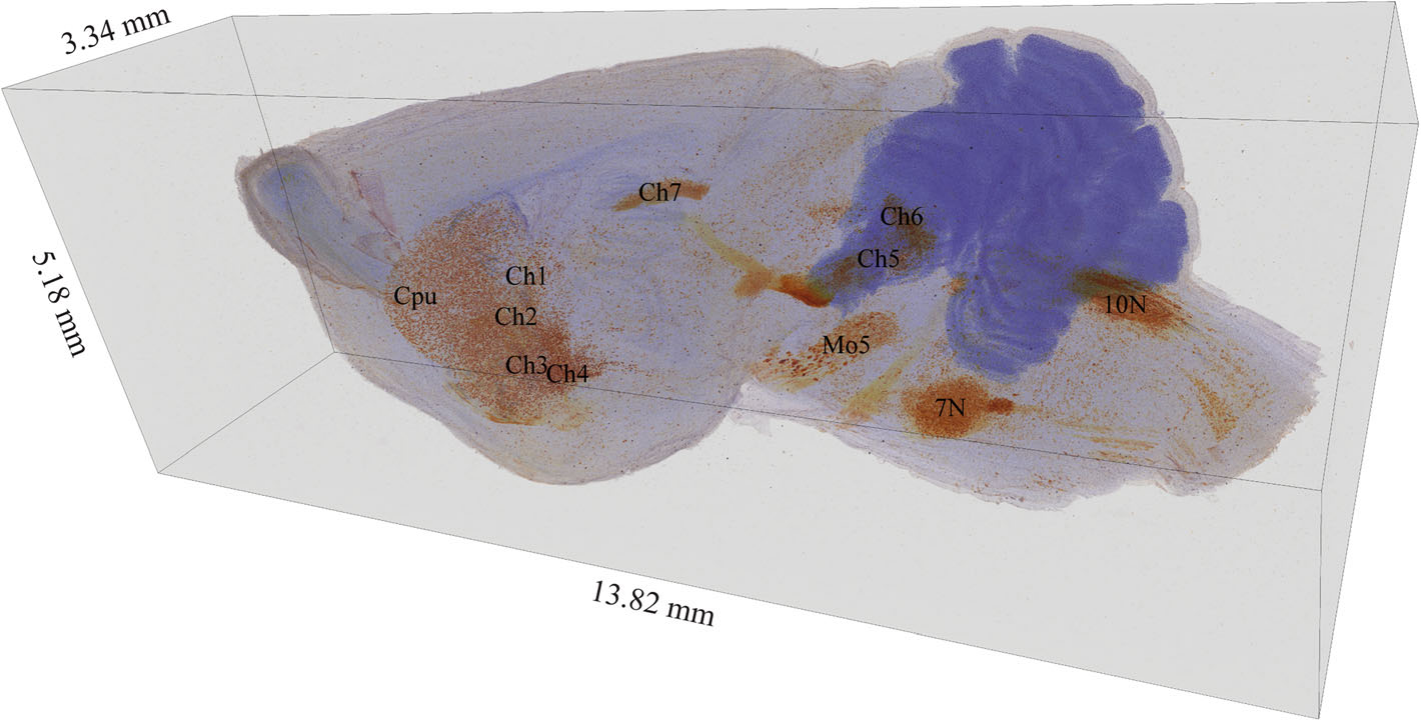
3D reconstructed image of cholinergic neuron distribution in the left hemisphere of the brain. In primates, cholinergic neurons are assigned to eight groups: Ch1 = medial septal (MS), Ch2 = vertical limb of the diagonal band of Broca (VDB), Ch3 = horizontal limb of the diagonal band of Broca (HDB), Ch4 = nucleus basalis of Meynert (NBM), Ch5 = pedunculopontine nucleus (PPN), Ch6 = laterodorsal tegmental complex (LDT), Ch7 = medial habenula (MH), Ch8 = parabigeminal nucleus (PBN). In this figure, Ch1–7 correspond to related cholinergic neuron groups in the mouse brain. Cpu = caudate putamen, Mo5 = motor trigeminal nucleus,7 N = facial motor nucleus, 10 N = dorsal motor nucleus of vagus

Most cholinergic neurons in the mammalian brain are found in four regions [34, 48, 49]: i.e., brainstem nuclei, subset of thalamic nuclei, striatum, and BF nuclei. We reconstructed a 3D image stack of cholinergic neurons in the entire left hemisphere of a mouse brain by choline acetyltransferase staining (Fig. 2) [34].

The BF contains at least three distinct populations of neurons (i.e., cholinergic, glutamatergic, and GABAergic) across its different regions. Cholinergic neurons of the BF are located in clusters from the olfactory tubercle to the rostral end of the lateral geniculate bodies. At the anterior pole of the BF, cholinergic neurons are located in the medial septal (MS) and diagonal band, which consists of a horizontal limb (HDB) and a vertical limb (VDB).

At the posterior pole of the BF, cholinergic neurons are found in the NBM [48]. There are striking interspecies differences in the anatomy of the NBM. According to comparative anatomical studies, the NBM in rodents is rudimentary and considerably interdigitated with the globus pallidus, whereas in non-human primates and humans the nucleus exhibits substantial development in size and differentiation from surrounding cell groups [50].

**Fig. 3:**
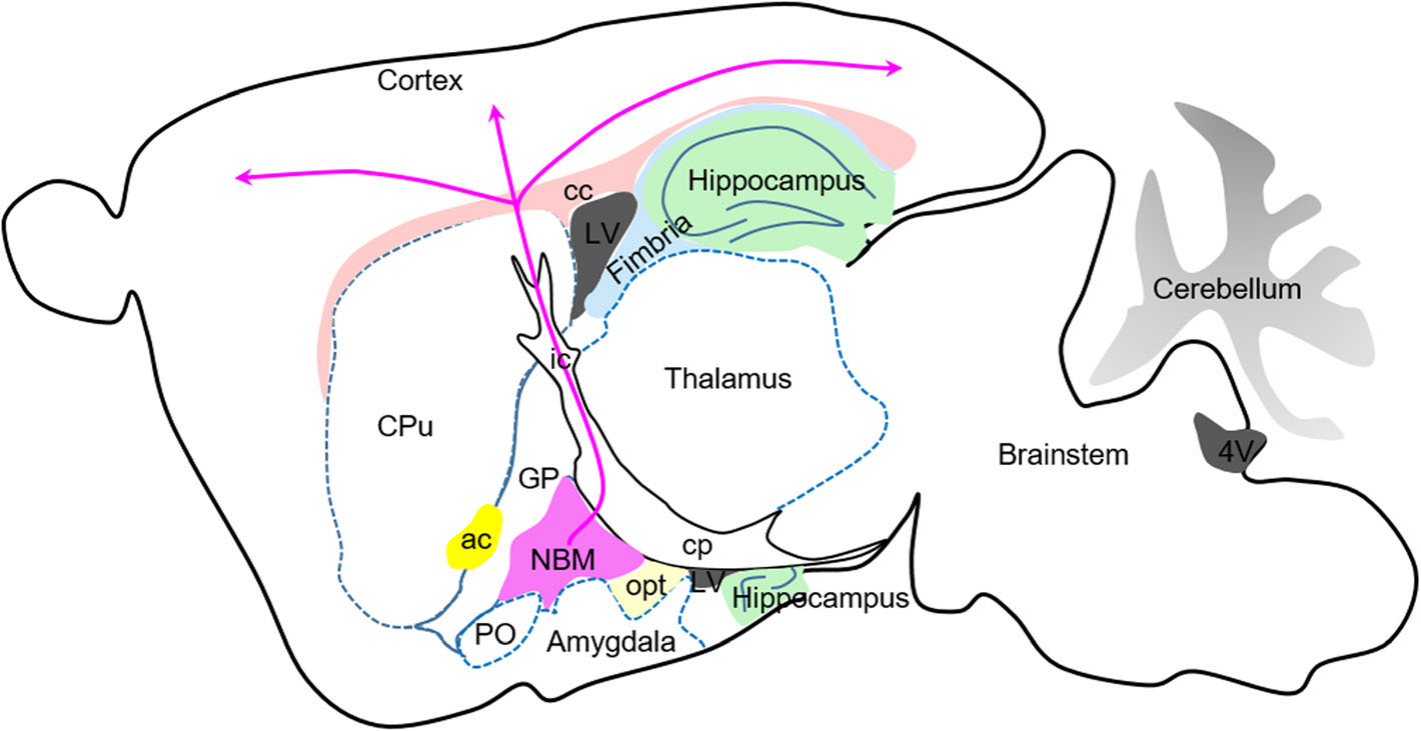
Sagittal schematic of NBM cell body location and projections in mice. NBM is extensive and has close topographical relationships with many different structures. Consistently, the NBM is always adjacent to the globus pallidus (GP) and is the main cholinergic projection to the neocortex and amygdala. Connectivity between individual sectors of the NBM and various cortical areas displays topographic specificity. Abbreviations: ac, anterior commissure; cc, corpus callosum; cp, cerebral peduncle; CPu, caudate putamen; GP, globus pallidus; ic, internal capsule; LV, lateral ventricle; opt, optic tract; PO, preoptic area

The anterior section of the NBM is limited inferiorly by the HDB, superomedially by the ventral globus pallidus, and superolaterally by the lateral extension of the anterior commissure [51]. The posterior section abuts the ansa lenticularis superiorly, the putamen laterally, the posterior tip of the amygdala inferiorly, and the optic tract medially [51] (Fig. 3).

The VDB is located along the ventromedial border of the septum. It contains the greatest number of hyperchromic fusiform neurons in the entire septal area [52]. These VDB cholinergic neurons merge with the MS cell group dorsally and with the HDB and NBM groups ventrally [52].

**Fig. 4:**
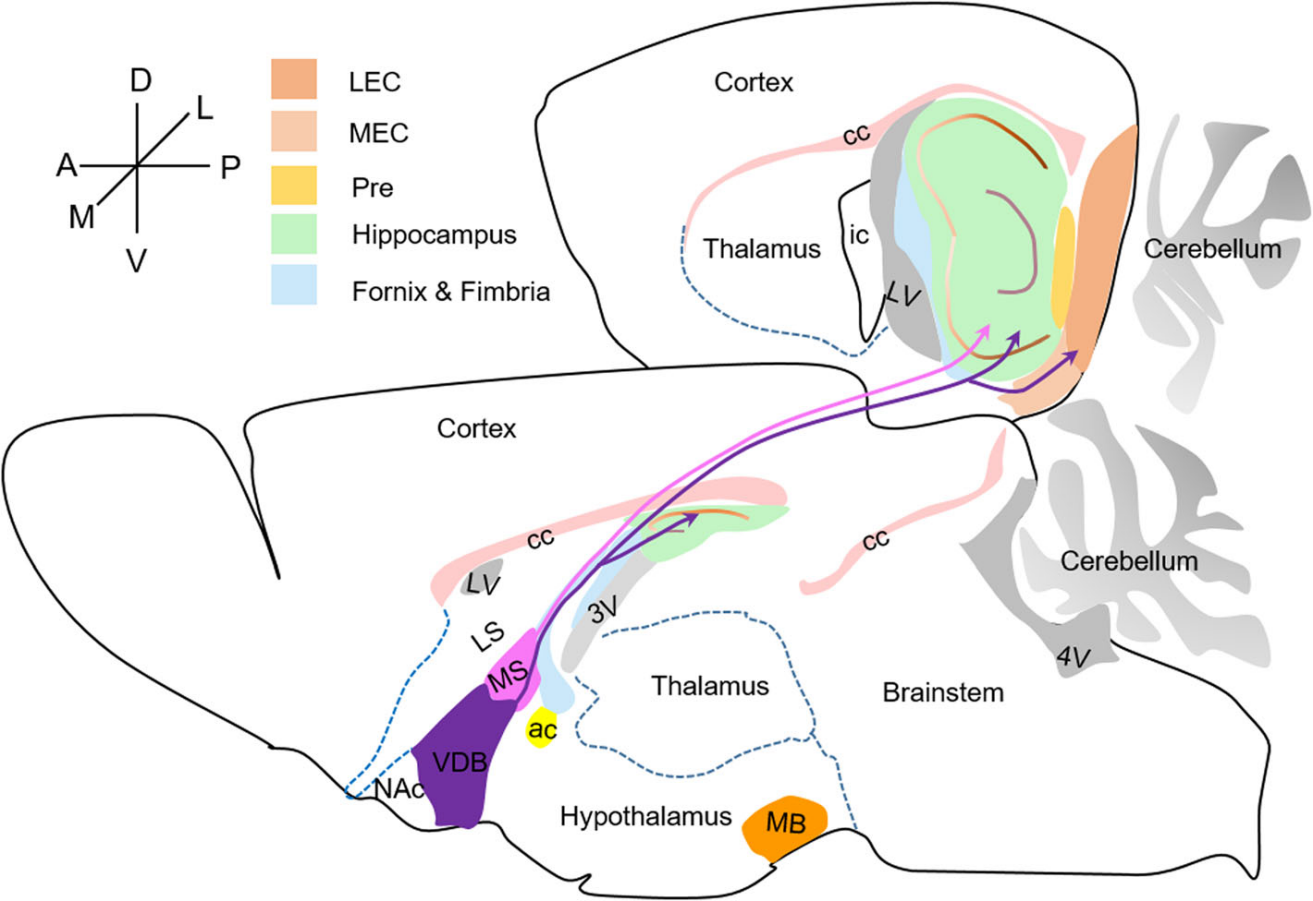
Sagittal schematic of locations and projections of MS and VDB in mice. The MS is located in the middle of the septum and many neurons are embedded among the precommissural fibers of the fornix. The VDB is located below the MS along the ventromedial and dorsomedial borders of the LS. The VDB projects via the fornix and fimbria to both the dorsal and ventral hippocampus formation, whereas the MS projects only to the ventral hippocampus. Abbreviations: ac, anterior commissure; cc, corpus callosum; ic, internal capsule; LEC, lateral entorhinal; LS, lateral septum; LV, lateral ventricle; MB, mammillary body; MEC, medial entorhinal; MS, medial septum; NAc, nucleus accumbens; Pre, presubiculum

These BF cholinergic projection neurons send extensive, multi-branched inputs to the neocortex, archicortex (e.g., hippocampus), and other subcortical structures [52, 53]. New tracing technologies have revealed that individual cholinergic neurons can project to distinct distal brain regions via different projection routes and that adjacent BF neurons can have highly diverse projection patterns [49]. The NBM constitutes the single largest source of cholinergic innervation to the entire cortical surface (Fig. 3), whereas the VDB is the major source of cholinergic innervation to the hippocampus from the basal cholinergic nuclei [52, 54] (Fig. 4). The CA1 pyramidal neurons and dentate gyrus (DG) in the dorsal hippocampus receive afferent cholinergic inputs almost exclusively from the VDB, whereas the corresponding cell layers in the ventral hippocampus are supplied by both the VDB and MS [55] (Fig. 4). The VDB projects to the hippocampus via the fornix and fimbria [56] and can directly innervate newly generated immature neurons in the DG of adult mice [18].

Retrograde tracing studies have shown that different BF subregions receive diverse inputs from multiple olfactory cortices [57]. Cholinergic neurons in the medial and caudal HDB and VDB receive inputs from the entire olfactory system, including the olfactory bulb, anterior olfactory nucleus, EC, basolateral amygdala, and especially the piriform cortex and hippocampus; whereas, the MS and part of the rostral HDB receive inputs predominantly from the hippocampus and the NBM receives inputs mainly from the central amygdala [57]. NBM neurons also receive other cortical input from the orbitofrontal cortex, anterior insula, temporal pole, entorhinal cortex, and medial temporal cortex [58], although most sensory, motor, and association areas in the frontal, parietal, occipital, and temporal lobes do not project to the NBM [58]. There are also subcortical inputs to the NBM from the septal nuclei, nucleus accumbens-ventral pallidum complex, and hypothalamus [58]. These synaptic inputs to the NBM consist of cholinergic, catecholaminergic, and γ-aminobutyric acid axons [59].

### Results of human DBS in memory circuitry for AD

#### Clinical trials of DBS in the medial temporal lobe

The potential of fornix-DBS (f-DBS) for memory improvement was first suggested by a case study reporting activation of autobiographical memories and improvement in verbal and visuospatial memories in a patient who underwent DBS for obesity [14]. To localize the stimulation site responsible for these changes, stereotactic coordinates of the active DBS contacts were determined from postoperative images. These coordinates were then plotted onto the Schaltenbrand-Wahren brain atlas and estimated to be adjacent to the columns of the fornix [60]. This discovery led to a phase I clinical trial consisting of six patients with early stage AD (ClinicalTrials.gov Identifier: NCT00658125) [61]. During implantation surgery, two subjects reported stimulation-induced autobiographical experiential phenomena. Memory improvements were also reported in some participants after 12 months of DBS, especially in those who experienced vivid experiential phenomena during surgery and had less severe memory problems at baseline [61]. Functional connectivity analyses also revealed that 1 year of f-DBS treatment increased cerebral glucose metabolism in two orthogonal networks: i.e., the frontal-temporal-parietal-striatal-thalamic network and frontal-temporal-parietal-occipital-hippocampal network [62]. Higher baseline metabolism in similar cortical regions prior to DBS and increased metabolism in similar regions after 1 year of DBS were correlated with better outcomes in global cognition, memory, and quality of life [62]. Among the six patients, bilateral hippocampal volume increases were observed in the two patients with the best clinical response, with one of these patients exhibiting preserved hippocampal volume 3 years after diagnosis [63]. In contrast, none of the matched control AD patients, who received only ongoing medication, demonstrated bilateral hippocampal enlargement [63]. This study demonstrates that in addition to modulating neural circuit activity, DBS can influence the natural course of brain atrophy in a neurodegenerative disease. Based on these phase I trial results, a randomized, double-blind, placebo-controlled, delayed-start phase II clinical trial (ADvance trial) was conducted at multiple North American institutions (ClinicalTrials.gov Identifier: NCT01608061) [64]. Across surgeons and treatment centers, the ADvance research group reported that it was feasible to accurately target DBS to the fornix without direct injury [64]. Bilateral f-DBS was also well tolerated by patients with mild probable AD at 90 days after surgery [64]. At 12 months after electrode implantation, the ADvance research group evaluated active “on” versus sham “off” bilateral DBS directed at the fornix in 42 patients with mild AD and found increased cerebral glucose metabolism without adverse effects [64]. Recently, although the ADvance research group reported no differences in cognitive outcomes among participants as a whole, participants aged ≥65 years appear to have benefited from DBS, whereas patients aged < 65 years appear to have worsened [65]. The ADvance trial is still active but not recruiting, so the influence of f-DBS on different AD stages is still unclear.

In 1934, Penfield first reported that an epileptic patient subjected to low intensity electrical stimulation to specific regions of the cortex during awake neurosurgery “re-lived” an event from his/her past; between the 1930s and 1960s, Penfield further reported 40 similar cases [66]. Since this pioneering work, other studies have also described experiential phenomena after electrical stimulation [67, 68]. Such findings are the major inspiration for preliminary trials aimed at improving memory performance using electrical stimulation in the EC. A group from the University of California implanted intracranial depth electrodes in seven subjects to identify seizure onset zones for subsequent epilepsy surgery and found that stimulation of the EC enhanced memory of spatial information applied during learning [67].

#### Clinical trials of DBS in the basal forebrain

Among the earliest targets for electrical stimulation in AD was the NBM. In 1984, attempting to augment the remaining tonic cholinergic inputs to the cortex and increase associated cortical metabolic activities, Turnbull et al. implanted DBS electrodes into the left NBM of a 74-year-old man with probable AD. After 8 months stimulation, the authors reported no clinically significant response, although they did not use detailed neuropsychological assessments with standardized measures [69]. Improvement in cognitive function by NBM-DBS was first demonstrated in a patient with slowly progressive Parkinson-dementia syndrome implanted with two NBM electrodes in addition to electrodes in the subthalamic nucleus [15]. This discovery led to a phase I clinical trial recruiting six patients with light to moderate AD (ClinicalTrials.gov Identifier: NCT01094145). Considering all limitations of this pilot study, DBS of the NBM was both technically feasible and well tolerated [70]. Patients who were less affected before treatment were less likely to deteriorate during chronic DBS [70]. Based on this observation, they performed NBM-DBS on two younger patients with milder AD [71] and found that changes in vitamin B12 compared to baseline levels following NBM-DBS were associated with cognitive status [72]. Furthermore, Alzheimer’s Disease Assessment Scale scores assessed over 24 months showed that NBM-DBS performed at an earlier stage of the disease and at a younger age may have a favorable impact on disease progression and cognitive function [73].

### Possible therapeutic mechanisms of DBS in memory circuitry

One possible therapeutic mechanism of DBS in the memory circuits is diminished synaptic loss. Surgical lesions of the descending postcommissural fornix or complete fornix both result in MB shrinkage without MB neuronal loss [74, 75]. We found that EC-DBS could rescue synaptic defects of the EC layer II to CA1 that were selectively degenerated in AD mice [76]. Tonegawa et al. found that optogenetic induction of long-term potentiation of the perforant path restored spine density from EC synapses to the DG in AD mice [77]. In addition, VDB-DBS has been shown to rescue the reduction in cholinergic synapse density in the DG [18]. These results indicate that diminished synaptic loss is feasible following DBS treatment.

Secondly, DBS in the memory circuits may also induce neurogenesis. Neurogenesis continuously occurs throughout life in the subventricular zone and granule cell layer of the DG. Adult-generated DG cells are thought to contribute to the formation of hippocampus-dependent memories [78]. The precommissural fornix contains cholinergic fibers innervating the DG [56], which can increase the survival of newly generated immature neurons in the DG [79, 80]. A study on the effects of 4-h acute stimulation to the fornix found no evidence for induced neurogenesis based on similar numbers of BrdU/NeuN double-labeled cells in the DG of both f-DBS and sham groups [81]. However, we observed prolonged lifespan for newly generated immature neurons without an increase in the total number of BrdU-labeled neurons when cholinergic fibers were stimulated [18]. Lozano and Frankland also reported that EC-DBS can influence cognitive function via activity-dependent regulation of hippocampal neurogenesis in AD mice [68].

Thirdly, DBS in the memory circuits may also enhance cerebral glucose metabolism. Glucose is the obligatory physiological energy substrate of the brain [82], and regional reductions in glucose utilization in the temporal lobe and posterior cingulate area are common features in early AD, as revealed by PET and single photon emission computed tomography [83, 84]. Numerous studies have indicated that neurons are inherently capable of activity-induced glucose uptake and glycolytic processing [85–87]. Widespread increases in glucose consumption are found during and immediately after neuronal activation [88, 89]. Many studies examining brain metabolism by blood oxygenation level-dependent (BOLD) fMRI or cerebral blood flow by PET have indicated a close relationship between DBS and its global neuromodulatory effects [90, 91]. For example, the increased metabolism observed in two orthogonal networks after 1 year of f-DBS was correlated with better outcomes in global cognition, memory, and quality of life [62]. Furthermore, electrical stimulation of the perforant path with continuous high-frequency (100 Hz) pulses generated BOLD responses in the hippocampus formation and in various target regions, i.e., medial prefrontal cortex, nucleus accumbens, and ventral tegmental area/substantia nigra region [92, 93]. In the earliest NBM-DBS case, increased cortical glucose metabolism was detected by fluorodeoxyglucose (FDG)-PET studies [27]. A subsequent phase I NBM-DBS trial using FDG-PET found a global increase of 2–5% in cortical glucose metabolism in three of four patients, which was most pronounced in the amygdala, hippocampal, and temporal regions [55].

Fourthly, DBS in the memory circuits may also drive interactions between medial and corticolimbic circuits, leading to a release of various neurotransmitters. Previous research has reported that f-DBS can activate the hippocampus and induce the release of acetylcholine (ACh) in this region [94] due to stimulation of the precommissural fibers (Fig. 1c), which primarily consist of cholinergic axons from the nucleus of the diagonal band that innervate the hippocampus [95]. For NBM-DBS, low-frequency stimulation (20 Hz) in rats [70], which resembles the physiological discharge rate of NBM neurons during motor activity in freely moving animals [96], activated ACh release from cortical terminals originating in the NBM [97]. Intermittent NBM stimulation can also improve working memory in adult monkeys by increasing cholinergic transmission, with the effect abrogated by either nicotinic or muscarinic cholinergic receptor antagonists [98]. In addition, f-DBS can also drive dopaminergic and glutamatergic transmission in anesthetized swine through the nucleus accumbens [99]. Postcommissural fibers of the fornix (Fig. 1c), including subiculum to MB projections, can regulate stress hormones following memory retrieval [100]. Understanding the exact identity of these transmitters and their sources is critical for clarifying the therapeutic effects of DBS. For example, degeneration of dopaminergic neurons at pre-Aβ-plaque stages contributes to memory deficits and dysfunction in reward processing [101]. A subset of nucleus accumbens medium spiny neurons receive inputs from the ventral hippocampus via the fornix, the activation of which increases the number of spontaneously active dopamine neurons in the ventral tegmental area [102]. Chronic stimulation of f-DBS may result in continuous activation of dopamine neurons in the ventral tegmental area; however, continued activation of dopamine neurons by DBS impairs verbal memory persistently across long periods of time [103]. Therefore, more direct evidence is needed to illustrate the exact relationship between its therapeutic effects and the transmitters or hormones released by DBS.

Fifthly, DBS in the memory circuits may also reduce Aβ plaque load of the projecting targets of the DBS site. Although the Aβ hypothesis of AD remains unproven, it largely remains defined by the central tenet that accumulation of Aβ, in a variety of forms, triggers a cascade that harms neurons and synapses [2]. In young AD transgenic mice, EC-DBS is reported to reduce Aβ plaque load in the dorsal hippocampus, prefrontal cortex, and amygdala [16]. These areas are all direct projection targets of the EC. In contrast, however, EC-DBS did not reduce Aβ plaque load in six-month-old AD mice, despite successful rescue in memory deficits [16]. This suggests that DBS may act through Aβ plaque-dependent mechanism only in early stage AD. Alternatively, Aβ plaque load reduction may not be the primary mechanism by which DBS reverses memory deficits. This is consistent with a number of observations including: 1) before Aβ plaque deposition, amnesia in AD mice is age-dependent [104–106]; (2) similar dissociations between memory loss and Aβ plaque load have been reported in AD patients [107]; and (3) immunotherapy against Aβ rescues memory deficits in AD mouse models without affecting Aβ plaque load [108].

Lastly, DBS in the memory circuits may also selectively stimulate M1 Ach receptors. Drugs that promote cholinergic signaling by blocking enzymatic degradation of ACh account for 75% of FDA-approved treatments for AD and these cholinesterase inhibitors have been shown to improve cognition in mild-to-moderate AD patients [109]. Metabotropic cholinergic responses are mediated by the five muscarinic ACh receptors subtypes (M1–M5). The distribution of these receptors alters with aging and progress of AD [110]. The localization of specific muscarinic ACh receptor subtypes in the hippocampus, and the brain in general, largely comes from the early work of Levey and colleagues [111, 112]. M1 ACh receptor expression is high throughout the hippocampus, particularly in the DG, and exhibits graded expression across the CA subfields, increasing from CA3 to CA1 and the subiculum. The M1 ACh receptor plays a crucial role in learning and memory and is closely associated with AD, and thus has long been postulated as a therapeutic target. Pharmacological stimulation of M1 ACh receptors exhibits advantages for cognitive improvement in AD patients [113, 114]; to date, however, no M1 ACh receptor agonist has been approved by the US FDA due to adverse side-effects. VDB-DBS may mimic the therapeutic effects of M1 Ach receptors in the DG but avoid the side-effects of general administration of M1 ACh receptor agonists.

## Conclusions

Early hypotheses on DBS mechanisms were obtained from studies on movement disorders, proposing that stimulation inhibited neuronal activity at the site of stimulation, thereby imitating the effects of surgical ablation. Subsequent studies have challenged this view, instead suggesting that high frequency stimulation increases and synchronizes output from the stimulation site by directly activating axons of local projection neurons, even if somatic activity near the DBS electrode is suppressed [115]. However, fundamental questions remain about the effects of DBS on the neurons surrounding the electrode and on their larger scale consequences. In AD DBS studies, increased glucose metabolism, neurotransmitter release, and neurogenesis suggest an activating effect of DBS at the neurocircuitry level.

Recent studies have also revealed network level effects of DBS. It appears that the stimulation targets that improve memory primarily function at the network level in order to coordinate activities among multiple brain regions. It is thought that different brain areas are involved in distinct stages of memory, with the hippocampus, especially the DG, essential for memory formation and the initiation of memory consolidation [116]. The earliest symptom of AD is anterograde amnesia, and the earliest pathological changes are observed in the EC and hippocampus. Therefore, DBS targets that directly rescue EC and hippocampal neurons may be most effective for slowing AD progression. However, the most intensively studied DBS targets in AD patients are the fornix and NBM based on serendipitous findings rather than knowledge of AD pathological mechanisms. These DBS targets appear to be related to memory retrieval [100] or fast modulation of conveyed cognitive information [117], rather than memory formation and consolidation. Moreover, these stimulation targets are chosen by different study groups based on the best outcome according to their own working hypotheses. For memory improvement induced by DBS, however, it is crucial that we determine whether these DBS sites have selective effects on specific types of memory and which locations are preferable for improving each. Several research groups have realized the above-mentioned limitations and started to compare effects between two different targets: i.e., the fornix and NBM. Two such prospective double-blind comparison studies will be conducted at Hospital San Carlos, Madrid (ClinicalTrials.gov Identifier: NCT03290274) and Xuanwu Hospital, Beijing (ClinicalTrials.gov Identifier: NCT03352739).

**Fig. 5:**
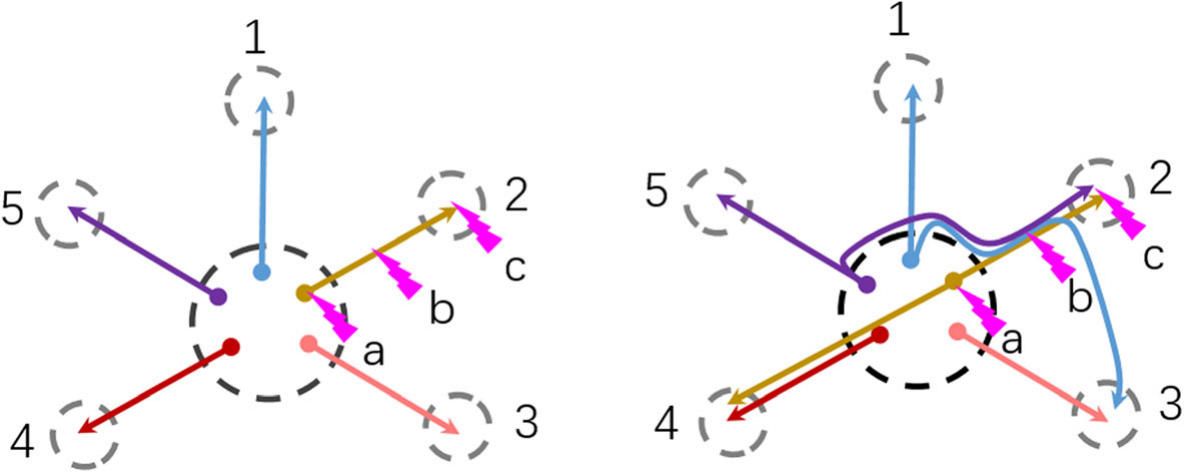
Schematic of different stimulation sites and activation effects based on different axon projection styles. Left, if projection style is ‘one area, one target’, there are no different activation effects in projecting to target 2 between the cell body, axon pathway, and axon terminal stimulation. Right, if the projection style is ‘one area, multiple targets’, there are many different activation effects between the cell body, axon pathway, and axon terminal stimulation. Stimulating the cell body may activate target 2 and 4, stimulating the axon pathway may activate target 2 and 3, and stimulating the axon terminal may activate target 2 only

Individual cortical neurons often innervate multiple target regions [118], therefore, the effects could be different when stimulating the axon terminal, axon pathway, or cell body (Fig. 5). A systematic review of the broad literature concerning electrical stimulation concluded that the qualitative nature of electrically evoked memories largely depends on the site of stimulation. Specifically, stimulation in the rhinal cortex usually induces personal semantic reminiscences, whereas hippocampal stimulation induces episodic memories [119]. In animal studies, it is not possible to distinguish semantic reminiscences from episodic memories; however, in light of the anatomical relationship between the EC and hippocampus, we postulate that EC-DBS mainly affects the hippocampus. Additional research is needed to clarify the EC-hippocampus effect and the differences in stimulation patterns between EC-DBS and f-DBS.

Furthermore, electrical DBS that targets a brain area or pathway cannot distinguish cell type and afferent source. Due to optogenetics, however, we can selectively activate a subgroup of cell bodies in one location or their axon terminal in one of their destination locations and compare the effects on target neuron activity or animal behavior. Nevertheless, it is still not clear whether we can directly translate the effects of cell type-specific or target-specific optogenetic DBS into electrical DBS, particularly as recent research indicates that astrocyte activity may also contribute to de novo neuronal potentiation and memory enhancement [120]. Furthermore, optogenetic stimulation in certain instances may not yield clinical benefits due to its high specificity, which can be a clinical disadvantage.

Although many clinical and animal studies of DBS have shed light on this as a possible new therapy for AD, many more studies are required to systematically evaluate the effects of electrical stimulation and its underlying mechanism.

## Data Availability

Not applicable.

## Abbreviations

ACh: Acetylcholine
AD: Alzheimer’s disease
Aβ: Amyloid beta
BF: Basal forebrain
DBS: Deep brain stimulation
DG: Dentate gyrus
EC: Entorhinal cortex
f-DBS: fornix-DBS
HDB: Horizontal limb of diagonal band
LEC: Lateral EC
MB: Mammillary body
MEC: Medial EC
NBM: Nucleus basalis of Meynert
PET: Positron emission tomography
VDB: Vertical limb of diagonal band
